# Clinical efficacy of pre-trained large language models through the lens of aphasia

**DOI:** 10.1038/s41598-024-66576-y

**Published:** 2024-07-06

**Authors:** Yan Cong, Arianna N. LaCroix, Jiyeon Lee

**Affiliations:** 1 School of Languages and Cultures, Purdue University, West Lafayette, USA; 2 Department of Speech, Language, and Hearing Sciences, Purdue University, West Lafayette, USA

**Keywords:** Predictive markers, Human behaviour

## Abstract

The rapid development of large language models (LLMs) motivates us to explore how such state-of-the-art natural language processing systems can inform aphasia research. What kind of language indices can we derive from a pre-trained LLM? How do they differ from or relate to the existing language features in aphasia? To what extent can LLMs serve as an interpretable and effective diagnostic and measurement tool in a clinical context? To investigate these questions, we constructed predictive and correlational models, which utilize mean surprisals from LLMs as predictor variables. Using AphasiaBank archived data, we validated our models’ efficacy in aphasia diagnosis, measurement, and prediction. Our finding is that LLMs-surprisals can effectively detect the presence of aphasia and different natures of the disorder, LLMs in conjunction with the existing language indices improve models’ efficacy in subtyping aphasia, and LLMs-surprisals can capture common agrammatic deficits at both word and sentence level. Overall, LLMs have potential to advance automatic and precise aphasia prediction. A natural language processing pipeline can be greatly benefitted from integrating LLMs, enabling us to refine models of existing language disorders, such as aphasia.

## Introduction

The advent of Large Language Models (LLMs) such as ChatGPT is progressively reshaping the landscape of clinical natural language processing (NLP) reserach^1–8^. These models often surpass previous NLP benchmarks, likely because they share computational principles with human language processing^9^. LLMs have shown the potential to predict, diagnose, and measure language disorders in persons with psychosis^8^ and dementia^10^. Yet, there is a limited understanding of the potential contributions and advancements that LLMs could bring to diagnosing language disorders such as aphasia. We aim to bridge this gap.

Aphasia is most often caused by a left hemisphere stroke. Aphasia impacts both language production and comprehension, making it challenging for persons with aphasia to communicate effectively and navigate daily life. Aphasia diagnosis involves comprehensive assessments by speech-language pathologists (SLP). The diagnosis typically relies on standardized tests such as the Western Aphasia Battery-Revised (WAB-R, Kertesz^11^), which includes paper–pencil tasks that generate accuracy scores on structured language tasks (e.g., picture description, object naming, repeating words and phrases). Collecting and analyzing a discourse sample of natural language production is a critical component to the diagnosis of aphasia, as they reveal specific impairments in speech fluency, grammar usage, word finding, and semantic coherence, above and beyond binary accuracy scoring. However, SLPs rarely incorporate quantitative discourse indices into clinical management of persons with aphasia^12,13^ because of time constraints and a lack of sufficient skills in how to quantitatively analyze discourse samples.

As such, developing automated programs for analyzing the natural speech of persons with aphasia has been a recent focus in clinical research. For example, a computerized language analysis software, CLAN (Computerized Language Analysis) has been developed to assess spoken discourse in persons with aphasia^12–18^. Software such as CLAN has the potential to facilitate clinical research and practice, since the coding and analysis can be (semi-)automated. However, this software appears to be used minimally among practicing SLPs as CLAN transcripts requires manually coding using a specific format for each analysis. These elaborate annotations can be tedious and lack consistency. Clinicians therefore need more streamlined and less resource intensive pipelines for discourse analysis, a gap LLMs can potentially fill.

LLMs have led to significant breakthroughs in NLP that may increase the feasibility of their clinical use. Purohit et al.^19^ explored ChatGPT in a *qualitative* text analysis, showcasing how prompt engineering can be used for word retrieval in aphasia. Salem et al.^20^ fine-tuned a LLM in Zaheer et al.^21^ to quantitatively predict paraphasic errors in speech produced by persons with aphasia. Ortiz-Perez et al.^22^ and Sanguedolce et al.^23^ both showed that OpenAI’s Whisper can be used in automatic speech recognition and transcription in aphasia. However, how to integrate recent LLMs into an NLP pipeline to automatically measure language deficits in persons with aphasia is understudied.

The recent literature in NLP and LLMs more broadly shows increased interest in *surprisals*, an index of negative log-probability of the occurrence of words in an utterance given preceding context^24–30^. In natural speech, speakers constantly select and assemble words in a linear order following language-specific rules. Because both lexical and grammatical properties of the context can influence the probability of the occurrence of the upcoming word, surprisals can be a useful metric to capture both word-level and structure-level impairments that are common in many persons with aphasia^5^. As illustrated in examples (1–3), each word’s surprisal is computed by GPT2 based on previous words. The whole utterance’s surprisal is the summation of all the words’ surprisals, divided by utterance length. With an utterance produced by a person in the healthy control group (example 1), GPT2 output a low surprisal score. By contrast, example (2) has a same-length utterance produced by a person with aphasia. Because the main lexical verb in (2) is missing after *let’s*, this utterance yields a high surprisal score. Similarly, in example (3), use of primarily noun phrases, leading to an impoverished syntactic structure, also leads to a higher surprisal score.

1. *and they get married and live happily ever after* (control; GPT2 surprisal score: 3.04)
2. *okay let's something there to get everybody around it* (aphasia; GPT2 surprisal score: 6.26)
3. *very nice, little girl and her bag and* (aphasia; GPT2 surprisal score: 6.86)

Computational linguistic studies show that LLMs-surprisals is a valid predictor of human real-time sequence-by-sequence processing times^26,31–39^, and it has been used in (psycho-)linguistic^40–45^ and morphosyntactic analyses^46^. On the other hand, Huang et al.^47^ and Amouyal et al.^48^ suggest that LLMs-surprisals cannot fully account for syntactic disambiguation difficulty and plausibility. Motivated by previous investigations in aphasia, NLP, and linguistics, we use LLMs surprisals to bridge LLMs and aphasia language analysis.

So far, use of LLMs computed surprisals in aphasia research is quite limited. Rezaii et al.^5^ (see also Rezaii et al.^49^) proposed that sentence surprisal, derived from GPT2, is a promising index to assess common sentence-level and word-level abnormalities in aphasia that are caused by syntactic processing deficits. Rezaii et al.^5^ specifically found that higher sentence surprisals were predicted by increased use of simpler sentence structures and the more frequent use of high informative (e.g., open class) than low informative (e.g., closed class) words. In addition, they showed that higher sentence surprisals in their patients with nonfluent variant of primary progressive aphasia correlated with common clinical features of agrammatism, including a higher open-to-closed class words, higher nouns-over-verbs, higher heavy-to-all verb ratio, and overuse of nominalized verb forms (-*ing*). While these findings are promising, further research is needed to more systematically evaluate the clinical efficacy of sentence surprisals in larger samples and across different aphasia types to further understand what aphasia deficits are captured by LLMs-surprisals.

The rapid development in LLMs enables surprisals computation to extend beyond the classic causal language modeling in GPT2. Novel architectures such as instruction tuning^27^ and sliding window attention^50^ have also been implemented and are gaining attention in NLP. Thus, there is a critical need to calculate surprisals with a more updated and systematic set of LLMs. Yet, there has been no systematic investigation of how recently developed LLMs perform in aphasia studies. Razaii et al.^5^ used GPT2 by Radford et al.^51^. Ghumman^52^ analyzed surprisals in stroke-induced aphasia, but they used the classic *n*-grams and neural sequence models rather than LLMs. Hence, in this study, we extend previous work by investigating more recent LLMs, and LLMs with different scales and architectural assumptions. We selected five GPT-type models so that we could investigate how scaling affects model prediction accuracy with the goal of providing a broader perspective on the capabilities and limitations of LLMs in a clinical context. We propose that a good understanding of LLMs structures would equip aphasia researchers with the knowledge to pinpoint the appropriate LLM, and scale it up if needed. Our model selection is also due to the observation that not every language biomarker researcher will have as much computational power as an industry practitioner, and larger LLMs do not always imply better language capacity. We therefore lay out LLMs’ linguistic sensitivities through a computationally accessible and streamlined NLP pipeline, with an attempt to demystify LLMs usage in aphasia research. Through this systematic investigation, we hope to aid future language disorder researchers in selecting the appropriate LLM for their purposes.

The purpose of this study was to evaluate the clinical efficacy of LLMs-surprisals as a suitable index for measuring deficits at the lexical-syntax interface in a large sample of patients with post-stroke aphasia, extending Rezaii et al.’s^5^ work in primary progressive aphasia. Specifically, we asked (a) if LLMs-surprisals can reliably predict the presence and subtype of post-stroke aphasia; and (b) using a series of analyses, we further sought to determine what aspects of language LLMs-surprisals may capture. To test these questions, we used the AphasiaBank archived data^16^ and calculated surprisals for people with and without aphasia using spoken discourse from written transcripts without manual annotation.

Our broader motivation was to further establish the theoretical and clinical basis for using LLMs-surprisal as a suitable index for aphasia discourse assessment. Clinically, there are two primary motivations. First, utilizing LLMs can help inform healthcare practitioners on identifying subtle language patterns that may not be captured by the existing tests, hence facilitating timely decisions of whether referral to a SLP is needed. Second, through subtyping, we can understand that surprisal can be used to capture distinctive deficits that are associated with different aphasia syndromes. This could provide useful information for SLPs in determining how to treat individual patients with aphasia.

## Methods

### Predicting the presence of aphasia

We first examined LLMs’ efficacy in diagnosing if a person has aphasia or not. Establishing LLMs’ efficacy in predicting the presence of aphasia is important for several reasons. First, it is computationally necessary to demonstrate LLMs basic sensitivity to the presence of aphasia before showcasing their ability to subtype the aphasias. Second, while an SLP is likely to know if someone has aphasia or not without computerized measurements, a nurse or doctor who initially interacts with the patient may not be as well versed in language disorders, especially when they are subtle. LLMs could be helpful in identifying which patients need to be referred to a SLP for a language evaluation. Further, many patients with mild aphasia report changes in their everyday discourse that are not captured by standardized tests such as the WAB-R that heavily rely on the accuracy of specific responses. Hence, LLMs may also aid SLPs in identifying people with latent aphasia who might also benefit from language therapy.

#### Data description

All discourse transcripts were drawn from the AphasiaBank^16^. One structured discourse task (story retelling narrative of the Cinderella story) was selected in a group of adults with aphasia (N = 441, age: mean 60.17; range 30–91; SD 10.95) and an age and sex matched control group (N = 341, age: mean 50.92; range 18–89; SD 21.38). During the Cinderella task, participants reviewed a wordless picture book of Cinderella for a few minutes. After that they were asked to tell the story of Cinderella without looking at the book. Both groups of participants were monolingual English speakers. To be sure that patients have a diagnosis of aphasia, we included only those with a WAB-R Aphasia Quotient (WAB-R AQ) less than or equal to 92.8 (mean 68.82; range 10.8–92.8; SD 17.64), the cut-off for diagnosing aphasia per the WAB-R^11^. The two groups were matched using the R *Matchit* package^53^. Considering the sample size, we specified the method parameter as “nearest” to implement nearest neighbor matching, using a logistic (probit) regression propensity score^54^. We provide detailed demographic information and group-wise numbers of observations in the supplementary (Table S1).

#### LLMs selection and surprisals calculation

Pre-trained autoregressive LLMs, like GPT2, were used to compute the new language index, surprisals. Such LLMs adopt causal language modeling, a pretraining task where the model reads texts in sequential order and needs to predict the next word^30^. These models are also called unidirectional LLMs, since the prediction is based on only the left-side of the current token^27^. This structure makes GPT-type LLMs more appropriate than other LLMs in surprisal calculation because it is compatible with the next word prediction pre-training task. Surprisal is the negative log-probability of a token or sequence of tokens given preceding context, as calculated by an LLM^30^. More formally, the surprisal of a target token T (current word w_*t*_) in a context C (previous words w_1…*t−*1_) was computed as Eq. (1). When w_*t*_ was tokenized by a LLM into multiple subword tokens, we took the average of the subword tokens probabilities.

1 $$Surprisal(T|C) = - logP(w_{t} |w_{1...t - 1} )$$

Derived from Eq. (1), we first computed surprisals at the utterance level: we summed the surprisal of the utterance over each token given the previous context, normalizing by the utterance length. We then computed surprisal at the paragraph level. We included approximately 33 utterances per aphasia participant (range [1,142], upper quartile (75%) = 45, SD = 24), and 47 utterances per healthy control participant (range [7,219], upper quartile (75%) = 56, SD = 31). Thus a “paragraph” could be a participant’s whole response to the Cinderella retelling task or just a subset of a response. Paragraph surprisal was computed by taking the mean over each utterance’s surprisal.

We included five open-sourced variants of GPT-type LLMs with a range of sizes. Since we need to derive surprisals from LLMs token-wise log probability rather than from LLMs generated (natural language) text, our method is called “direct probing”. This means we can only select LLMs that are open-sourced, so that they expose individual token’s log-probability^55^. LLMs selection is also motivated by our intension to examine how scaling would influence LLMs’ capacity: GPT-2 with 124 million parameters^51^; DistilGPT-2 with 82 million parameters^56^, trained as a student network with the supervision of GPT-2; and GPTNeo with 1.3 and 2.7 billion parameters^57,58^, henceforth GPTNeo-1.3B and GPTNeo-2.7B, which is close to the size of the smallest models in the GPT-3 family. We are aware of the rapidly evolving landscape of LLMs. Therefore, we included Mistral with 7 billion parameters (v0.1, henceforth Mistral-7B^50^). Similar to GPT-4, Mistral-7B also uses causal language modeling and next token prediction in its pre-training, and it only contains the decoder part of the transformer. Mistral-7B outperforms the popular Llama2-13B on all the widely used benchmarks^50^. It is also one of the largest and latest autoregressive LLMs that is open-sourced. To operationalize LLMs derived metrics, we used *minicons*^59^, an open-source utility that provides a consistent API for behavioral analyses of LLMs. All the LLMs used in this study are hosted in HuggingFace (https://huggingface.co/models; as of May 2024).

#### LLMs input format

The input for each LLM was orthographically transcribed text as we are interested in investigating how effective LLMs are in diagnosing aphasia in a clinical setting where clinicians have minimal to zero time to conduct quantitative analysis of discourse samples. Also, indices without manual coding enables reproducibility and consistency, since elaborate manual coding are prone to errors and inter-coder inconsistencies. Here are examples illustrating what LLMs input look like versus a program (CLAN software) that requires elaborate coding. According to CHAT format used in CLAN software, “& = laughs” marks non-speech verbalization such as laughter, and “[+ exc]” marks extraneous comments such as “wait a second”. These codes tell the software to not include the associated utterances in the analysis. Thus, a CHAT annotated transcription looks like *&* = *laughs I haven’t really had an injury. So [*+ *exc] luckily I haven’t had any injuries* but the program is actually analyzing *I haven't really had an injury. so luckily I haven't had any injuries*. In contrast, LLMs can handle the latter input, *I haven’t really had an injury. so luckily I haven’t had any injuries,* which requires minimal annotation from human annotators. Verbatim transcription or elaborate annotation such as “& = laughs” and “[+ exc]” are not needed in LLMs input. For the current research, our LLMs-based NLP pipeline does not include an automatic step for speech-to-text transcription. Instead, we focused our analyses on text and not sound features as LLMs are mainly pre-trained on text data. We argue that using a LLM pre-trained in sound data such as OpenAI’s Whisper would be more appropriate when sound features are involved in benchmarking LLMs’ clinical competence.

#### Model construction and optimization

Four different machine learning classification models were constructed and optimized using LLMs-computed surprisal features as predictor variables to predict whether a given text is produced by the control or the aphasia group. The four models include decision tree, random forest, gradient boosting, and support vector machine classifier (SVM). Decision trees split the feature space into smaller regions based on feature values, while random forests aggregate predictions from multiple decision trees. Gradient boosting builds decision trees sequentially to correct errors, and SVM finds the hyperplane that best separates classes in the feature space. The aim here is to systematically examine LLMs’ clinical efficacy with different classification models, and to demonstrate how evaluation metrics may change with different types of classifiers. To further tease apart the distinct contributions of each LLM to model efficacy, we constructed and optimized five separate models using the classification method with the best efficacy in predicting the presence of aphasia from our main analysis.

In order to reduce the risk of overfitting and to balance the datasets, we first split the entire dataset into the training (2/3 of the whole data) and the gold testing datasets (1/3 of the whole data). Using the training dataset, we conducted nested *k*-fold cross-validation with the hyperparameters optimization algorithm *grid search*. Since our whole dataset is small, we set *k* (inner and outer) as 3, and we focused on tuning only the essential hyperparameters. We chose nested cross-validation to address the overfitting concern^60^. With nested cross-validation, hyperparameter search and tuning should have a lower chance to overfit the dataset because it is exposed only to a subset of the dataset provided by the outer cross-validation procedure. We then tested the tuned models’ performance on the gold testing dataset, which has never been used in training or validation. In other words, we evaluated each model’s prediction performance on the gold testing dataset, and model’s classification report is based on the gold testing dataset. Our machine learning models selection strategies are inspired by Cawley and Talbot^60^. SHAP (SHapley Additive exPlanations) values were visualized to reveal feature importance in models’ classification. Detailed search space and model configuration procedures are given in the supplementary (Table S3-S4). All the machine learning models were constructed and evaluated using scikit-learn^61^.

### Predicting aphasia subtypes

We next examined LLMs’ efficacy in subtyping the aphasias. This was to increase our understanding of what aspects of LLMs-surprisals may be capturing clinically. For example, non-fluent aphasia (e.g., Broca’s aphasia) and fluent aphasia (e.g., Wernicke’s and Anomic aphasia) differ in terms of their sentence level linguistic deficits. In Broca’s aphasia, morphosyntax is more impaired while semantics are more impaired in Wernicke’s aphasia. These impairment differences should give rise to different LLM-surprisal patterns, which should aid in subtyping the aphasias. Further, subtyping analysis will reveal how clinically effective the proposed aphasia classification methods are. Additionally, we maintain that different subtypes of aphasias may have different treatment needs. Thus, it is critical to classify them for more precise and personalized treatment.

#### Data description

The same dataset, discourse task, and inclusion criterion were used to select a group of adults with aphasia who had one of three subtypes of aphasia: Broca, Wernicke, and Anomic (N = 186). This selection had two primary motivations. First, these three subtypes, especially Broca’s and Wernicke’s aphasia, show distinct linguistic impairments, which will inform what LLMs-surprisals are characterizing in a clinical linguistics setting. Second, these three subtypes of aphasia are the most widely available and frequently investigated for studies using the AphasiaBank^62^. To balance the data points, we used the *Matchit* package with the same parameters setting as the first dataset. We randomly sampled 2200 unique observations for each subtype of aphasia. In total, there were 6600 unique observations. This is the largest amount of aphasia-subtype-balanced unique observations we can get from 186 participants. Detailed demographic information and subtype-wise number of observations are given in the supplementary (Table S2).

#### LLMs details

The same LLMs, surprisals calculation methods, and input format used to predict the presence of aphasia were also used to subtype the aphasias. To examine whether surprisals are providing new or existing information about aphasia, we calculated models that included just LLM-surprisals and compared those to models with language indices commonly used in existing aphasia research (hereafter referred to as the existing indices; described below) and to models that include both existing language indices and surprisals. This type of analysis was not included in the aphasia presence analysis, because that analysis was a baseline proof-of-concept task that showcased LLMs potential for clinical efficacy. We view aphasia subtyping as the main examination of how much clinical efficacy LLMs have.

#### Combining the existing language indices

Only the language indices that did not require manual annotation in CLAN were included as existing language indices. We elected to remove language indices that required verbatim manual coding and elaborate annotations, for example, utterance error, percentage of word error, and so on. This is because such indices involve verbatim and elaborate manual annotation, which can be time consuming and inconsistent across clinicians and SLP raters. This decision was also made in part to be consistent with the text input we used for the LLMs.

The selected language indices reflect three broad categories that are commonly used by clinicians and computational studies of aphasia^12–16,52,62–66^. First, indices of linguistic productivity or fluency included mean length of utterance and number of utterances in the sample. Second, the index of lexical diversity was type-token ratio (number of unique lemmas divided by the number of total running lemmas). Third, indices of syntactic complexity included the ratio of open to closed words (open class words divided by closed class words), sentence complexity ratio (number of clauses divided by number of sentences), nouns over verbs (number of nouns divided by number of verbs), nouns to prepositions ratio (number of nouns divided by number of prepositions), verbs ratio (number of verbs divided by summation of verbs and nouns), nouns percentage (number of nouns divided by number of words), verbs percentage (number of verbs divided by number of words), adjectives percentage (number of adjectives divided by number of words), adverbs percentage (number of adverbs divided by number of words). These language indices were computed using NLTK^67^ and spaCy based automatic text analysis tools *TAACO*^68,69^ and *TAASSC*^70^.

#### Model construction and optimization

The same four machine learning classification models used to predict the presence of aphasia were constructed and optimized for subtyping the aphasias. We created three models to help parse apart whether LLMs-surprisals are adding new or existing information about language in aphasia: model (a) uses both LLMs-surprisals and the existing language indices, model (b) only uses surprisals, and model (c) only includes the existing language indices. The same hyper-parameter tuning and nested *k*-fold cross-validation methods used in the presence of aphasia analyses were implemented here. We again used SHAP values to visualize feature importance. We fit the *SHAP* explainer with the best configured model on the gold testing dataset.

In contrast to predicting the presence of aphasia, aphasia subtyping is a multi-class classification task. The three classes include Wernicke’s, Broca’s, and Anomic aphasia. We reported one versus one (one-vs-one) classification results. Unlike one-vs-rest that splits the data into one binary dataset for each class, the one-vs-one approach splits it into one dataset for each class versus every other class. In order to investigate how different strategies influence models’ prediction, we additionally constructed and optimized a random forest classifier using one-vs-rest approach. We created models that leverage each LLM separately and then compared model evaluation metrics, in order to understand how surprisals from different-sized LLMs may have an impact on model’ subtyping performance. Detailed search space and model configuration procedures are given in the supplementary (Table S5–S7).

### What do LLM-surprisals represent clinically?

We investigated the relationships between LLM-surprisals and existing language indices using two approaches. In the first approach, we built correlational models looking at the relationship between LLMs-surprisals and aphasia severity, and LLM-surprisals and the existing language indices outlined above. To do this analysis, we used the same aphasia dataset that was used to predict aphasia presence (N = 441). The same inclusionary criteria and sampling methods were used to select participants with aphasia. The same set of existing language indices used in aphasia subtyping was applied for building the correlational model. These existing language indices in this dataset were calculated using the same text analysis tools *TAACO*^68,69^ and *TAASSC*^70^. Surprisals were derived from LLMs, using the same methods and settings as in the aforementioned tasks. The alpha level in this paper is set as 0.05. Correlation effect size was considered strong if the coefficient was 0.5 or larger^71^. Statistical analyses were conducted in R^72^.

In the second approach, we examined surprisals between Broca’s and Wernicke’s aphasia to test the hypothesis whether surprisals can differentiate nonfluent aphasia from fluent aphasia. A healthy control group was also included as a comparison to further understand the surprisals metric. The aphasia dataset was the same one we used to predict aphasia subtypes (N = 186). We additionally selected a matched control group (N = 76) using the *Matchit* package. Our baseline comparison for this analysis was comparing Broca’s and Wernicke’s aphasia on two syntactic complexity indices (proportions of nouns over verbs, and proportions of clauses over sentences). These indices were used in a similar surprisal analysis^5^, which showed higher nouns over verbs as a meaningful marker of non-fluent aphasia. Rezaii et al.^5^ additionally found a nonlinear relationship between sentence-level surprisals and syntax frequency (i.e., the average correct use of syntactic rules). Note, Rezaii et al.^5^ used nouns over verbs and syntax frequency. Here, we take clauses over sentences as a proxy index to Rezaii’s syntax frequency index, because “clauses over sentences” has been shown to be informative in the subordination amount, which is a commonly used and recommended index of productive complexity^73^.

## Results

### LLMs’ efficacy in predicting the presence of aphasia

#### Leveraging all LLMs features at once

Table 1 includes the accuracy (percentage of correct predictions made by the model), precision (the model’s performance at classifying positive observations), recall (how “sensitive” the classifier is at detecting positive instances), and F1-score (a harmonic mean of the precision and recall) for the different machine learning classifiers. Each machine learning classifier has five predictors (i.e., surprisals calculated from each of the five LLMs). Overall, all four machine learning models showed good performance in predicting the presence of aphasia from surprisals, with relatively high F1-scores ranging from 0.84 to 0.92. Of these models, SVM was the best model, having an accuracy of 0.92, which suggests excellent model performance. In the medical field, machine learning metrics, specifically F1 scores, exhibit values spanning from 0.66 to 0.96^74^, where a value close to 1 represents good precision and recall values.

**Table 1:** Evaluation metrics of different machine learning classifiers in predicting the aphasia and the control group, with all five LLMs as predictor variables.

Machine learning classifier	Accuracy	Precision	Recall	F1-score
Decision tree	0.86	0.87	0.86	0.86
Random forest	0.84	0.84	0.84	0.84
Gradient boosting	0.86	0.86	0.86	0.86
SVM	0.92	0.92	0.92	0.92

**Figure 1:**
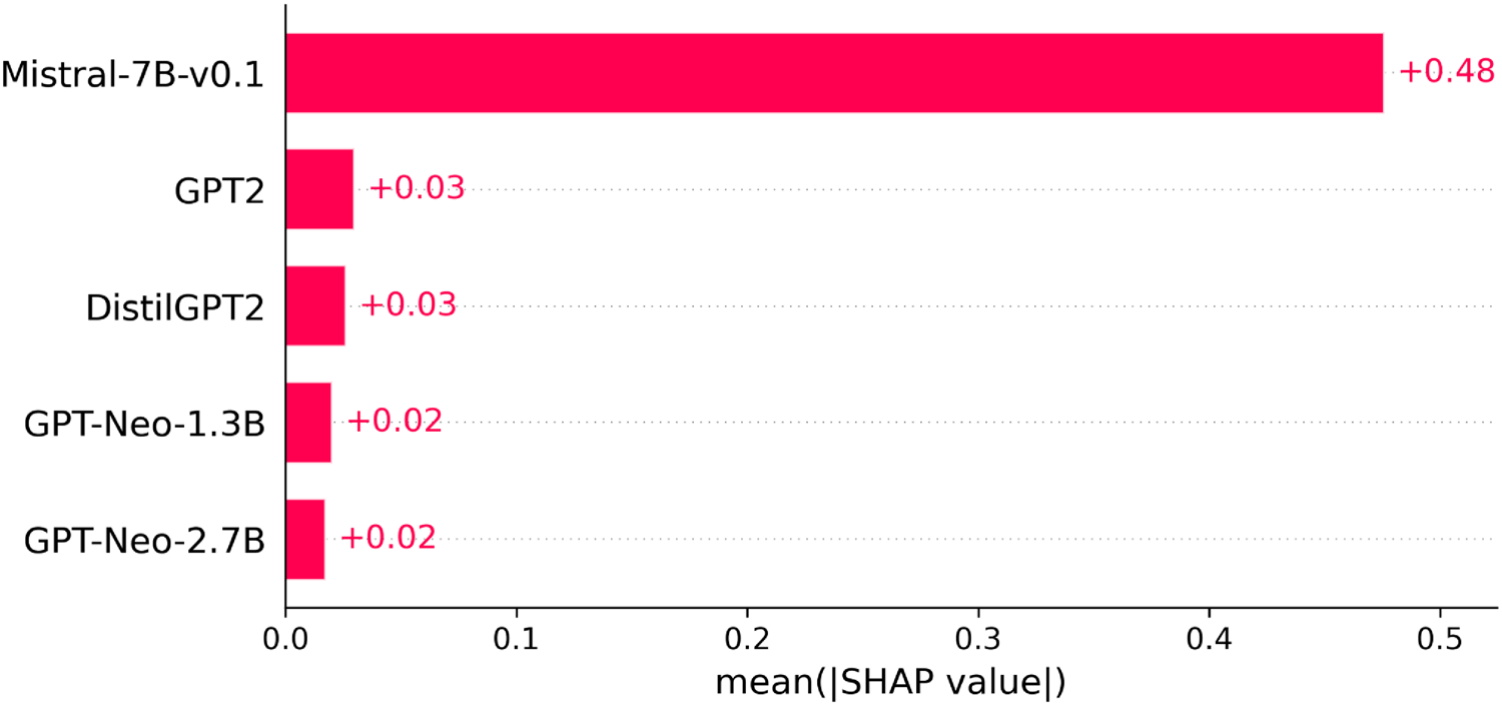
LLMs features importance on SVM predicting the presence of aphasia, rank based on the absolute SHAP value.

We next visualized how each LLM’s surprisal feature contributes to SVM prediction using the SHAP value, since SVM showed the best efficacy (c.f., Table 1). As shown in Fig. 1, the most decisive surprisal feature in predicting the presence of aphasia came from Mistral-7B. Mistral-7B uses a novel architecture (i.e., sliding-window attention)^50^ and is significantly larger than the other LLMs included in this analysis. However, it is likely that Mistral-7B’s novel architecture, not its size, is driving its superior performance as the smaller LLMs, GPT-2 and DistilGPT-2, showed comparable results as the larger GPTNeo-1.3B and GPTNeo-2.7B models.

#### Leveraging one LLM at a time

To further examine each LLM’s contribution to the model, we additionally constructed a SVM with one predictor variable at a time. We chose SVM, because it gave the best efficacy in predicting the presence of aphasia among the four machine learning classifiers (c.f. Table 1). Evaluation metrics are reported in Table 2.

**Table 2:** Evaluation metrics of SVM in predicting the aphasia and the control group, with individual LLM as the predictor variable.

One predictor variable at a time	Accuracy	Precision	Recall	F1-score
SVM (Mistral-7B)	0.88	0.89	0.88	0.88
SVM (GPTNeo-2.7B)	0.84	0.85	0.84	0.84
SVM (GPTNeo-1.3B)	0.86	0.89	0.86	0.85
SVM (DistilGPT-2)	0.76	0.84	0.76	0.74
SVM (GPT-2)	0.71	0.82	0.71	0.69

Table 2 suggests that Mistral-7B individually led to the best SVM in predicting the presence of aphasia (F1-score 0.88), which is in line with the SHAP value rank (Fig. 1). This indicates that Mistral-7B can excel with and without other LLMs features in aphasia presence detection. Mistral-7B is followed by the two GPTNeo LLMs. The smaller LLMs DistilGPT-2 and GPT-2 showed lower efficacy (F1-scores 0.69) than larger LLMs, suggesting that surprisals derived from small LLMs should be interpreted with caution when used as individual variables predicting the presence of aphasia.

### LLMs’ efficacy in predicting aphasia subtypes

#### Leveraging all LLMs features at once

Evaluation metrics are given in Table 3. This table shows that overall, adding LLM indices on top of the existing language indices improved the gradient boosting (GB), SVM, and random forest (RF) classifiers’ accuracy, precision, recall, and F1-score, but had limited impact on the decision tree (DT) classifier’s prediction efficacy. More specifically, adding LLMs to existing language features using the SVM classifier gave rise to the best overall prediction efficacy: the SVM F1-score changed from 0.73 (model c.) to 0.79 (model a.). This suggests that LLMs optimized with gradient boosting, SVM, and/or random forest classifiers are providing new information about aphasic language, and in conjunction with existing language indices, have the potential to advance automatic subtyping of aphasia, leading to decent efficacy.

**Table 3:** Evaluation metrics of different machine learning classifiers in predicting aphasia subtypes, with three different feature combinations.

	LLMs and existing indices (model a.)	LLMs (model b.)	Existing indices (model c.)
DT accuracy	0.65	0.53	0.68
DT precision	0.68	0.58	0.67
DT recall	0.65	0.53	0.68
DT F1-score	0.63	0.54	0.67
RF accuracy	0.76	0.61	0.76
RF precision	0.78	0.64	0.76
RF recall	0.76	0.61	0.76
RF F1-score	0.75	0.62	0.76
GB accuracy	0.71	0.6	0.69
GB precision	0.72	0.61	0.72
GB recall	0.71	0.6	0.69
GB F1-score	0.71	0.6	0.68
SVM accuracy	0.79	0.66	0.73
SVM precision	0.81	0.69	0.73
SVM recall	0.79	0.66	0.73
SVM F1-score	0.79	0.65	0.73

DT, decision tree classifier; RF, random forest classifier; GB, gradient boosting classifier; SVM, support vector machine classifier.

**Figure 2:**
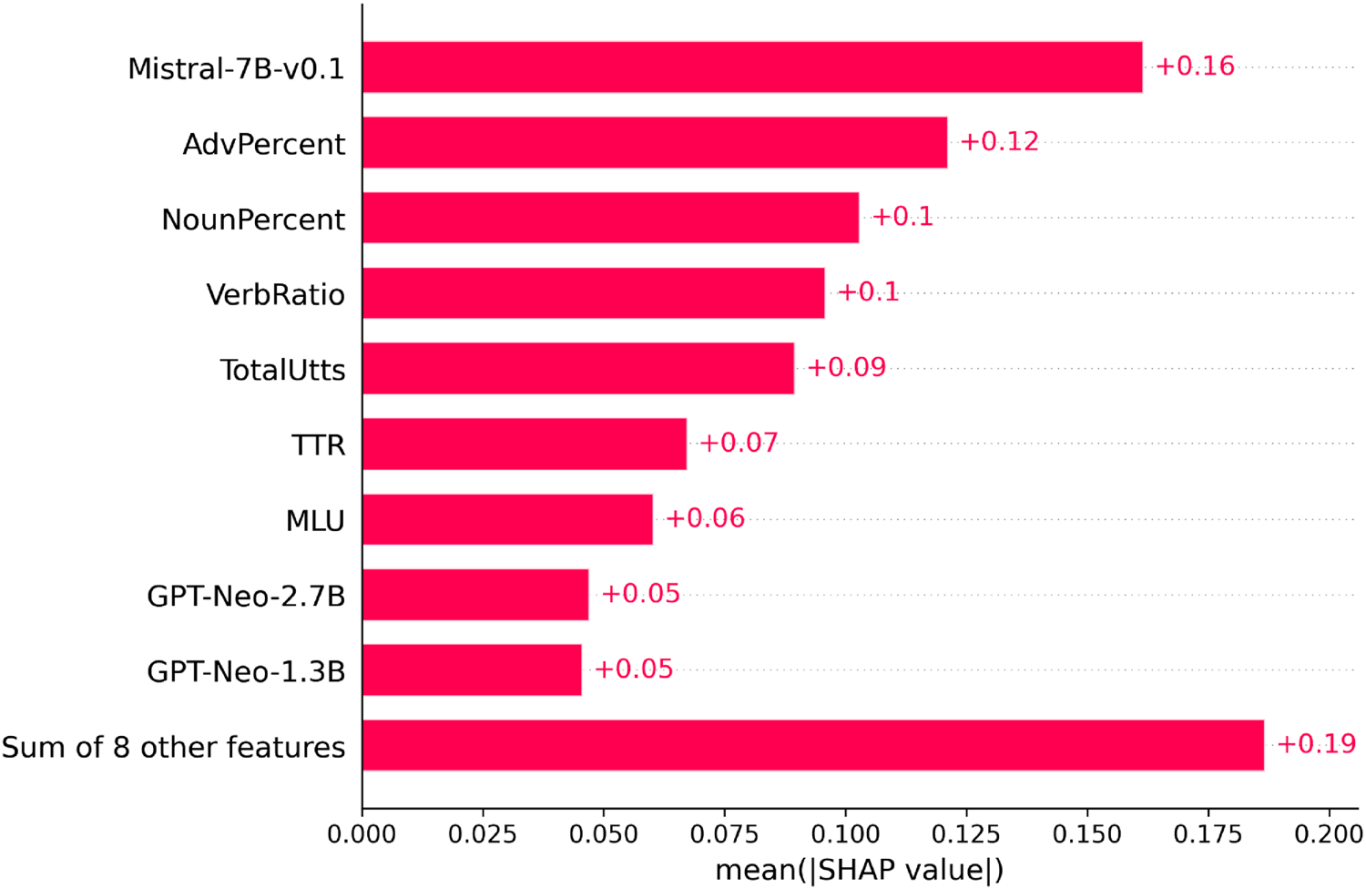
LLMs features importance on SVM predicting the subtypes of aphasia, rank based on the absolute SHAP value.

We next ranked the most informative features in predicting the aphasia subtypes based on the SHAP values from the best configured model (SVM) in Fig. 2. This allowed us to further quantify the contributions of LLM-surprisals versus the existing language indices to aphasia subtyping.

As shown in Fig. 2, for a SVM combining all predictor variables (Model a.), Mistral-7B showed the highest absolute SHAP value, followed by the existing language variables (c.f. Methods Sect. 2.2.3): AdvPercent (adverb percentage), NounPercent (noun percentage), VerbRatio (number of verbs divided by summation of verbs and nouns), TotalUtts (total number of utterances), TTR (type token ratio), and MLU (mean length of utterance). The two GPTNeo LLMs were less decisive with subtyping the aphasias. The “Sum of 8 other features” included GPT-2 and DistilGPT-2 surprisals, SentComplexity (sentence complexity ratio: number of clauses divided by number of sentences), VerbPercent (number of verbs divided by number of words), AdjPercent (number of adjectives divided by number of words), NounVerb (number of nouns divided by number of verbs), OpenClose (open class words divided by closed class words), and NounPrep (number of nouns divided by number of prepositions). Individually, these eight features have minimal impact on the model's prediction of the aphasia subtypes. The complete SHAP figure listing all these features is given in the supplementary (Figure S1). Overall, Fig. 2 suggests that surprisals calculated with Mistral-7B affect model prediction the most and separately from the existing language features, though the existing language variables are still robust and informative in subtyping the aphasias. These results also indicate that the smaller LLMs, GPT-2 and DistilGPT-2, are less capable of aphasia subtyping than the larger LLMs.

**Figure 3:**
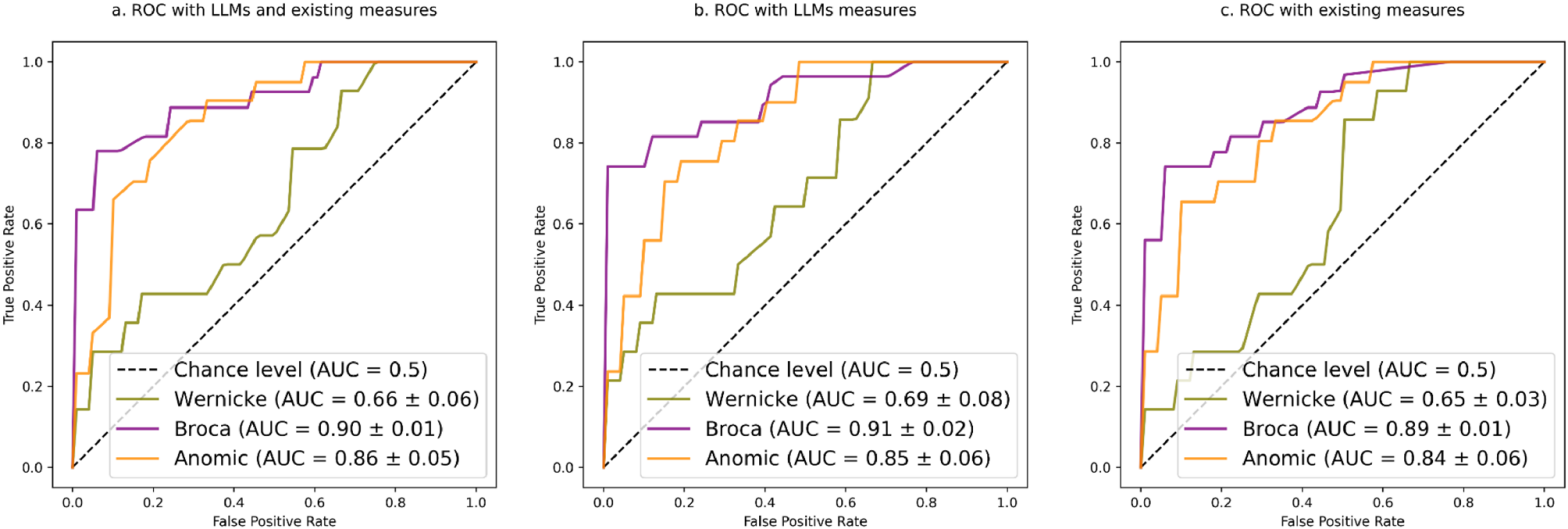
Multiclass Receiver Operating Characteristic (ROC) and area under the curve (AUC) metrics to evaluate the quality of the random forest classifiers One-vs-Rest, with LLMs and existing language indices (model a.), only LLM indices (model b.), and only the existing indices (model c.).

Additionally, to show a different classifier’s prediction efficacy with a different approach, Fig. 3 visualizes random forest classifiers predicting three aphasia subtypes in a “one-vs-rest” format: Wernicke’s versus Anomic and Broca’s, Broca’s versus Anomic and Wernicke’s, and Anomic versus Broca’s and Wernicke’s in the gold testing dataset. We implemented this one-vs-rest approach in random forest instead of SVM, although SVM seems to be the overall best classification method in our main analyses. This is because of random forest classifiers’ relatively better computational efficiency, ease of implementation, and robust performance across a wide range of datasets without the need for extensive parameter tuning. Considering the small size of the gold testing dataset, we implemented a stratified two-fold cross-validation. The mean and standard deviation of the AUC (area under the curve) of the model across both folds are reported. Three models (a,b,c) showed similar efficacy.

Figure 3 demonstrates that LLM indices (model b.) are the most effective in predicting Broca’s versus the rest and Wernicke’s versus the rest. Combining LLMs and the existing indices (model a.) leads to a more effective model in predicting Anomic versus the rest. The existing language indices (model c.) are slightly less effective than LLMs (model b.) in teasing apart aphasia subtypes. Overall, using one versus the rest approach gives rise to better model performance than the one versus one approach (c.f. Table 3), at least for the random forest classifiers. Critically, both approaches reveal that adding LLMs features on top of the existing ones can improve a model's efficacy, suggesting that surprisals may represent a distinct aspect of language processing from existing clinical language indices. Further, we infer that there is intellectual and clinical merit to use a LLM index of a certain linguistic operation (e.g., surprisal) in advancing classic aphasia subtypes toward more precision-medicine.

#### Leveraging one LLM at a time

In order to examine individual LLM’s contribution to each model’s prediction and to avoid potential feature redundancy across the various LLMs-surprisal metrics, we created SVM for each LLM surprisal feature in Table 4, in a similar format as in Table 2. Interestingly, when using one LLM feature at a time to subtype aphasia (model b.), the smaller LLMs, GPT-2 and DistilGPT-2, showed higher F1-score than the larger ones such as Mistral-7B. When combining the existing features and one LLM feature at a time (model a.), we found that the smaller LLMs such as GPT-2 showed similar efficacy to Mistral-7B. A comparison of model a. in Table 2 where *all* LLMs surprisals are included to model a. in Table 4 where *individual* LLM features are included suggests that model a. in Table 4 does not excel further than model a. in Table 2. This suggests that including all LLMs-surprisals features at once as opposed to including one at a time helps optimize models’ configuration. This also indicates that surprisals calculated with different LLMs may not all represent the same aspect(s) of language.

**Table 4:** Evaluation metrics of SVM in predicting aphasia subtypes, with individual LLM features, one at a time.

	LLM and existing indices (model a.)	LLM (model b.)
Mistral-7B accuracy	0.76	0.63
Mistral-7B precision	0.77	0.49
Mistral-7B recall	0.76	0.63
Mistral-7B F1-score	0.76	0.55
GPTNeo-2.7B accuracy	0.74	0.65
GPTNeo-2.7B precision	0.75	0.54
GPTNeo-2.7B recall	0.74	0.65
GPTNeo-2.7B F1-score	0.74	0.57
GPTNeo-1.3B accuracy	0.74	0.53
GPTNeo-1.3B precision	0.75	0.64
GPTNeo-1.3B recall	0.74	0.53
GPTNeo-1.3B F1-score	0.74	0.55
DistilGPT-2 accuracy	0.74	0.69
DistilGPT-2 precision	0.74	0.59
DistilGPT-2 recall	0.74	0.69
DistilGPT-2 F1-score	0.74	0.62
GPT-2 accuracy	0.76	0.63
GPT-2 precision	0.77	0.66
GPT-2 recall	0.76	0.63
GPT-2 F1-score	0.76	0.62

### What do LLM-surprisals represent clinically?

#### Relationship between LLMs and existing language indices

**Figure 4:**
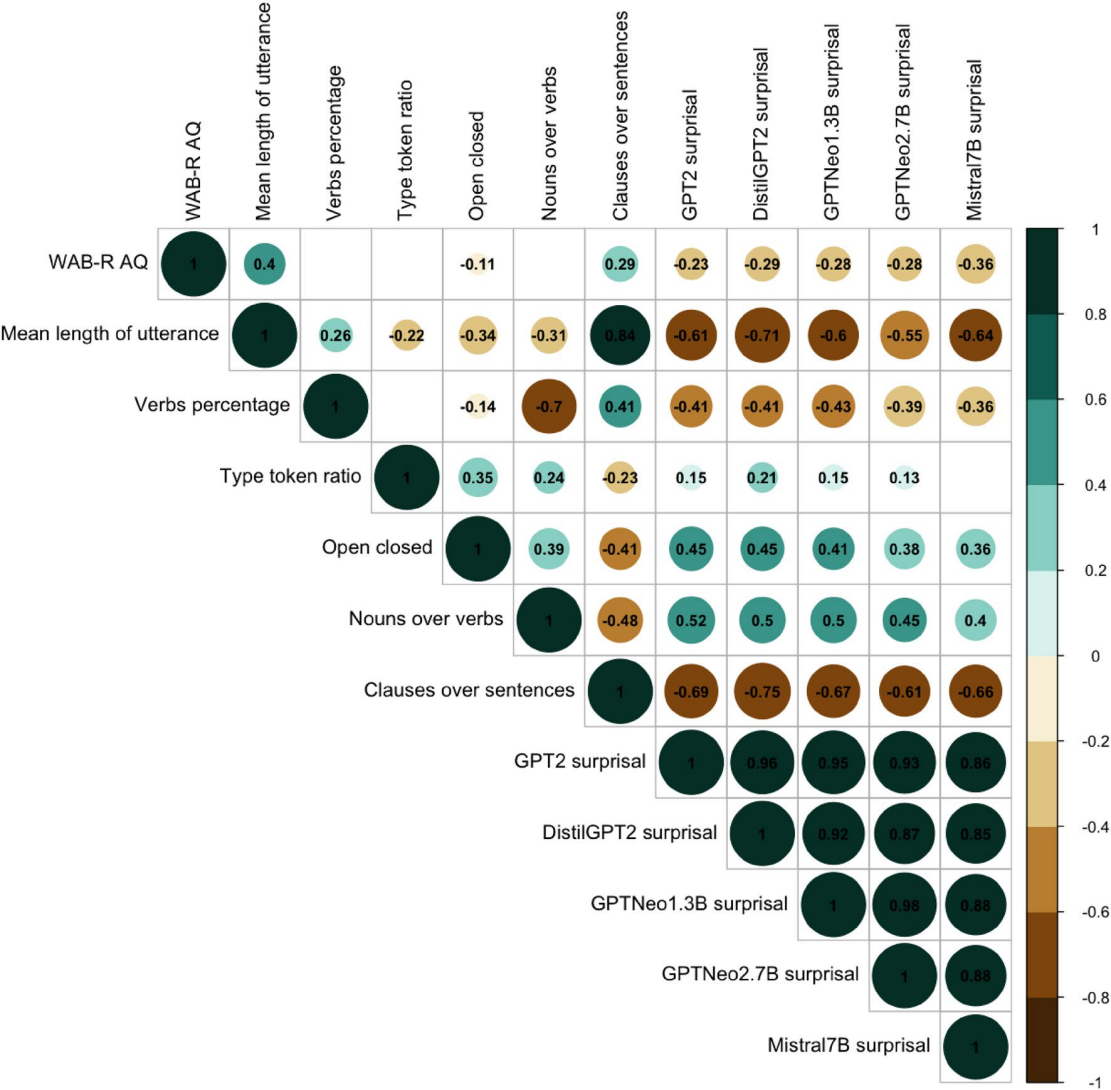
Correlations coefficients heatmap of LLMs-surprisals, WAB-R AQ, and selected existing language indices. Insignificant cells are left blank.

To better our understanding of what LLMs-surprisals are measuring in aphasia studies, we investigated how surprisals relate to aphasia severity and the existing language indices. Figure 4 shows Spearman correlation coefficients for LLMs mean surprisals, aphasia severity measured using the WAB-R AQ score, and commonly used language measures indices in aphasia^66,75^.

First, all LLMs-surprisals had a significant negative correlation with WAB-R AQ, indicating that patients with milder aphasia symptoms had lower mean surprisal scores. Mistral-7B, the largest LLM, showed the strongest correlation coefficient. This speaks to Mistral-7B’s architecture being better able to capture the severity of the aphasia impairment than the other LLMs. Second, all LLMs-surprisals were strongly negatively correlated with mean length of utterance. This suggests that a higher mean surprisal is an indicator of lower linguistic productivity and fluency, reflected in reduced length of utterances. Third, LLMs-surprisals and verbs percentage were negatively correlated, suggesting that reduced use of verbs is associated with higher surprisals. Fourth, all but Mistral-7B showed moderate *positive* correlations with type-token-ratio. This indicates that higher surprisals were associated with higher lexical diversity. Further, all the LLMs-surprisals are positively correlated with nouns over verbs, suggesting that overusing nouns and underusing verbs can be associated with higher surprisals. Moreover, all the LLMs showed strong negative correlation with the syntactic complexity index “clauses over sentences”. This implies that higher surprisals are associated with fewer embedded clauses and more limited syntax. All the LLMs-surprisals were also positively correlated with the ratio of open and closed class words. This reveals that overly relying on open class words with limited production of closed class words is related to higher surprisals, across LLMs.

Moreover, we found strong positive correlations within LLMs-surprisals. This is not surprising, because all the LLMs are unidirectional with only the decoder part of a transformer. Although these LLMs differ in size and specific architectures, they share the core architecture and pre-training task (causal language modeling, next word prediction). We would predict that the relation of these LLMs should be stronger, compared to the relation between a masked language model such as BERT and a causal language model like GPT-2. Clinically, we infer that using decoder unidirectional LLMs leads to consistent findings. Strong correlations within LLMs also imply that our finding is generalizable to any LLMs pre-trained with causal language modeling. Depending on specific research goals, we can focus on one LLM if computation resource is too limited to operate multiple LLMs.

#### Behavior of LLMs-surprisals in nonfluent versus fluent aphasia

To demystify specifically how LLMs behave in aphasia subtypes, and to further our understanding of what LLMs-surprisals capture, we conducted a three-way comparison across two aphasia subtypes—Broca’s and Wernicke’s aphasia, and the control group. We additionally carried out this comparison on two commonly used indices of syntactic complexity, proportions of nouns over verbs and proportions of clauses over sentences^5,73^, as a further way to understand what surprisals represent.

**Figure 5:**
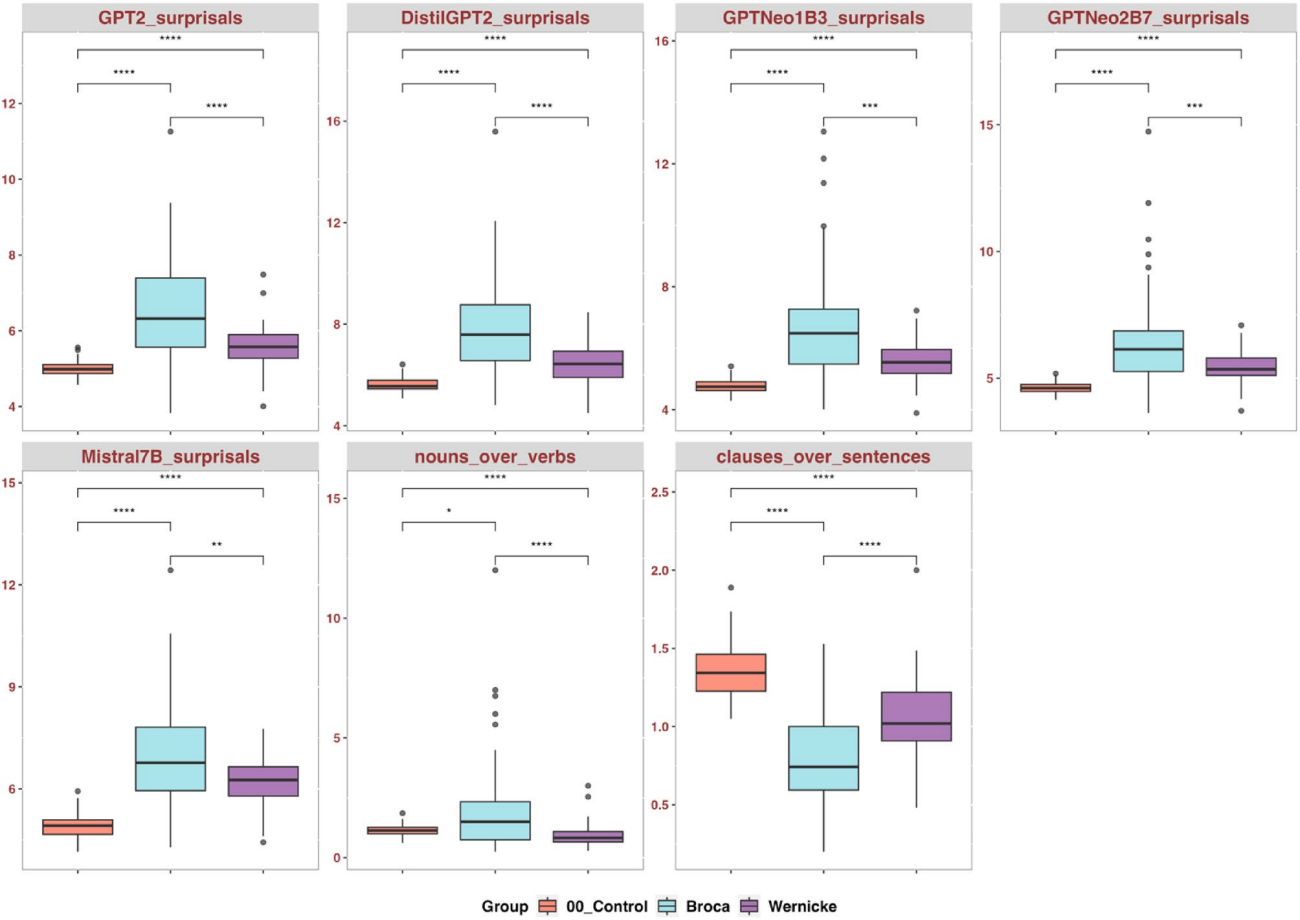
Wilcoxon tests with Bonferroni correction, comparing LLMs-surprisals behavior in non-fluent aphasia (the Broca’s aphasia group), fluent aphasia (the Wernicke’s aphasia group), and the Control group. Notation: ns: *p* > 0.05; *: *p* <  = 0.05; **: *p* <  = 0.01; ***: *p* <  = 0.001; ****: *p* <  = 0.0001.

Visual inspection of histograms and statistical examination (Shapiro–Wilk test, *p* < 0.05) indicate that the normality assumption is violated. Therefore, we used the non-parametric Wilcoxon test. As expected, compared to Wernicke’s aphasia, persons with Broca’s aphasia showed higher *nouns over verbs* and lower *clauses over sentences*, indicating reduced syntactic complexity in non-fluent aphasia (Fig. 5). Importantly, Wilcoxon tests across all the LLMs showed significant mean differences between those with Broca’s and Wernicke’s aphasia, with higher surprisals in the Broca’s aphasia group. These results echo the correlational model in Fig. 4, suggesting that agrammatic features of aphasia can be captured in LLMs-surprisals at both word and sentence level.

Across LLMs, the Control group showed lower surprisals than the fluent and non-fluent aphasia groups. This further justifies LLMs-surprisals’ general validity. Boxplots also showed noticeably larger interquartile range in the aphasia groups (especially the non-fluent aphasia), relative to the control group, suggesting more variability in aphasia.

## Discussion

This study examined the efficacy of LLMs in characterizing and predicting aphasia, an acquired language disorder. We aimed to answer two main research questions. First, relative to the existing diagnostic indices and tools, how can LLMs advance automatic prediction, measurement, and subtyping of aphasia? Second, what features of aphasia deficits do LLM-surprisals capture? Correlational and predictive machine learning models were proposed and evaluated. We found that NLP pipelines integrating LLMs show decent performance in classifying persons with aphasia and healthy controls, and with careful configuration, it can lead to higher efficacy in subtyping aphasia than a pipeline without LLMs. We additionally found that LLMs-surprisals relate to and complement the existing language measures.

### Advantages and disadvantages of LLMs usage in aphasia research

#### Advancements and challenges

LLMs provide several advantages: efficacy, efficiency, and scalability. First, our findings suggest that an NLP pipeline integrating LLMs enables testing and refining of language disorder models. LLMs features lead to excellent performance in diagnosing persons with aphasia and healthy controls. Adding LLMs indices improved the models’ accuracy in predicting subtypes of aphasia. Second, it is worth highlighting that the machine learning models with LLM features managed to differentiate between Broca's and Wernicke's aphasia since they represent non-fluent and fluent aphasias, respectively, even though we did not code for fluency in our input. This suggests that adding LLMs features on top of existing language indices enables more precise and effective models. Third, with CLAN, elaborate speech disfluency annotations can be time-consuming. We built all the models without any speech error or disfluency annotations. Reproducing a model based on CLAN features requires systematic annotation following certain conventions such as CHAT, whereas LLM based models can advance the performance without using verbatim or richly annotated datasets. Fourth, a more efficient pipeline can be more scalable, because it involves fewer operational steps and provides more automation. If a larger LLM is needed, we can easily take a pre-trained LLM off-the-shelf without further training or fine-tuning. Such versatility and flexibility in scaling can greatly facilitate scientific discovery with lower costs.

#### Scaling and its implication

We did not always find outstandingly higher efficacy using larger-scale LLMs. Although Mistral-7B and larger LLMs showed higher efficacy than smaller ones in classifying aphasia and healthy controls (Table 2, Fig. 1), we found the inverse when classifying subtypes of aphasia (Table 4). This leads us to critically examine the view that larger size LLMs can be superior to their smaller counterparts^47,76–78^. It is worth reconsidering the supremacy of larger LLMs. Our approach and results indicate the need for a more nuanced way in assessing LLMs’ efficacy for aphasia research. One of the primary limitations of larger LLMs is the immense computational resources required for their training and deployment. The massive number of parameters and computational power necessary for training these models makes them inaccessible to many researchers. Scaling massive LLMs also suffers from transparency decrease, as there is limited understanding of how LLMs’ abilities change as they scale up^78^. With the appropriate experimental design and dataset selection, smaller LLMs can outperform larger ones, and can provide better interpretability due to their simpler architectures^79,80^.

Why did the larger scale LLMs such as Mistral-7B not always give us significantly better measures? Our findings suggest that in subtyping aphasia, machine learning models involving larger scale LLMs underperform those involving smaller LLMs (Table 4). This result also indirectly aligns with Oh and Schuler^81^ and Shain et al.^38^. They found that larger scale LLMs show a worse fit to human reading times. Shain^37^ suggests that large scale LLMs with instruction tuning may “contaminate” the interpretability of next word prediction, hence they may lead to worse alignment with human behavior. We infer that this is likely to be a domain adaptability problem. Larger LLMs’ performance can deteriorate when applied to specific domains or dataset, for instance, an aphasia corpus, which large LLMs have not encountered during their pre-training. We indirectly hypothesize that smaller LLMs, on the other hand, because of their reduced architecture complexity, are more likely to exhibit better performance and adaptability in domain-specific datasets^82^. Our findings motivate us to argue that although large-scale LLMs have remarkably pushed the boundaries of NLP, their deployment comes with non-negligible trade-offs, for example, computational power requirements, lack of interpretability, and generalization limitations^80^. The finding that larger LLMs did not excel further justifies that, clinical researchers can achieve their goals by solely taking a smaller LLM off-the-shelf and conducting inferencing such as computing mean surprisals.

To address LLMs’ disadvantages and make the most of their advantages, we propose to fine-tune LLMs on a larger aphasia corpus, and test LLMs’ performance on a larger healthy control corpus. If LLMs still derive surprisal scores in the same pattern, it would validate LLMs derived metrics. For the time being, we used pre-trained LLMs without any form of controlling their source training data, parameters, or pre-training tasks. We argue that our approach improves the ecological validity of LLMs in aphasia research, making our whole pipeline accessible and generalizable in practice. As a showcase of methodology, we hope to introduce these versatile models, with attempts of exploring interpretability strategies. For next steps, we plan to further explore and validate LLMs derived metrics from fine-tuning perspectives.

### Interpret LLMs-surprisals in a clinical context

#### Clinical interpretation and applications

Taken together, the current findings support a clinical potential of LLMs-surprisals in predicting and understanding aphasia. LLMs sentence surprisals differentiated discourse speech produced by persons with aphasia and healthy adults and improved the models’ accuracy to differentiate common aphasia types. These findings suggest that in considering how speakers select and assemble words into sentences, an index such as surprisals can be effective in detecting pathologies associated with aphasia. As a meaningful biomarker, LLMs-surprisals can enhance clinical trials for latent aphasia or the “subclinical” group, namely persons who self-report aphasia but are not diagnosed as aphasia with WAB-R AQ. For this group, the existing language indices alone may not be sufficiently precise to help clinicians and SLPs make the decision. LLMs may become relevant. The existing indices in conjunction with LLMs-surprisals will greatly facilitate the process of effective clinical decisions. Better subtyping of aphasia would also be beneficial to understand why some patients with certain types of aphasia respond to certain language related treatments while other aphasia subtypes do not.

Our second set of findings further revealed that surprisals can be a useful index for detecting core agrammatic features that manifest at both the word and sentence-level^5^. The two levels of processing (use of high frequency content words and reduced syntax) are not modular, instead, patients with agrammatic aphasia use such strategies to maximize their communication within limited processing resources^5,49,83^. LLMs-surprisals, as a holistic index, integrate word and sentence level features, which may characterize aphasia patients’ communication patterns in ways that may not be salient when classic language indices are used. Our correlational analyses revealed that a range of traditional features of nonfluent agrammatism showed stronger associations with higher surprisals, compared to general aphasia severity (WAB-R AQ) or lexical diversity (type token ratio) measures. More specifically, clinical measures that are thought to reflect reduced fluency and impoverished structural complexity, including reduced mean length of utterance, reduced clauses over sentences, reduced production of verbs percentage were associated higher surprisal scores. Patients’ increased reliance on open-closed class words (higher open to closed class ratio) and nouns rather than verbs also led to higher surprisals. Lastly, the group of persons with Broca’s aphasia showed significantly higher surprisals compared to those with Wernicke’s aphasia, further confirming that surprisals reliably capture nonfluent agrammatic characteristics in aphasia. Overall, our findings refine previous studies in that surprisal captures the word-level and structural-level abnormalities in patients with aphasia experience, with greater sensitivity for agrammatic features.

Our LLMs based NLP pipeline has ecological validity. This is in line with previous studies on clinical applications of LLMs^84–86^. Our showcase reveals that we can gain meaningful information from LLMs using our laptop. Without computationally intensive tasks like training and fine-tuning LLMs, or labor-intensive tasks like elaborate annotation of a transcript, it is still feasible for clinical researchers to compute sufficiently sensitive metrics such as mean surprisals. We hope our methodology can inspire a wider application of LLMs in clinical practice. Moreover, our study can hopefully inspire the development of new methods to improve aphasia treatment. Integrating state-of-the-art NLP systems has the potential to improve accuracy and efficiency of aphasia prognosis and treatment, quantifying which language learning mechanisms in aphasia lead to greater improvement in language recovery, hence advancing the development of refined models for aphasia rehabilitation.

#### Computational interpretation

From the perspective of machine learning models, including LLMs-surprisals together with the existing language indices can advance models’ efficacy greater than a model with only LLMs-surprisals or the model with only the existing language indices. Another finding is that, although LLMs-surprisals are more decisive than some of the existing indices in subtyping aphasia, a model with existing indices outperformed a model with LLMs, regardless of using all LLMs at once or using one LLM at a time. This is possibly because next word prediction within a sentence, a pre-training task shared by all the LLMs in our experiments, is not sufficient to capture the complex and subtle linguistic patterns in aphasia. LLMs-surprisals can complement the existing language features^47^.

Note, LLMs-surprisals failed to surpass the existing indices only in the one-vs-one classification approach (a binary classifier is trained for every pair of classes). With a different classification task one-vs-rest (a binary classifier is trained for each class against all other classes combined), we found that LLMs slightly outperformed the existing indices (Fig. 3). Although both one-vs-one and one-vs-rest are multi-classification approaches, with three classes (Wernicke, Broca, and Anomic), misclassification is presumably more likely to occur with one-vs-one than with one-vs-rest. We stipulate that noise from misclassified cases in a one-vs-one approach potentially may skew the performance metrics^60^.

### Future directions

NLP research considers LLMs-surprisals as an index of *plausibility* and *relatedness*^41^, besides syntactic complexity, fluency features, and lexical properties^5,41–43,46,49,66,87,88^. We leave its clinical relevance for future justification. To what extent LLMs-surprisals can be a sensitive index of semantic plausibility or relatedness in a clinical context is open to discussion. For future studies, with larger sample size and boarder aphasia population, we plan to quantify how much LLMs-surprisals can capture nuanced differences between low-plausibility sequences and *extremely* low-plausibility ones. It is also possible that LLMs driven indices can go above and beyond, for example, accounting for discourse flow and topic complexity. For the current investigation, we focus on sentence as a measurement unit, and derive paragraph indices from sentential measures. It is likely that expanding context window sizes can lead to different findings.

The current study lays the groundwork for future studies to obviate not only manual coding but also transcription, achieving total automation in aphasia severity measurement. Although it can be cost-effective not having to conduct *verbatim* transcription, such as annotating speech errors and laughter, our pipeline is not completely automatic and still needs transcription. Given that our focus here is on LLMs that are pre-trained using mainly text data, our pipeline does not include LLMs-based speech to text transcription. With ongoing advances with automated text-to-speech transcription for impaired speakers and LLMs pre-trained on sound data, future research should replicate current findings with recorded speech samples to obviate transcription^22,23,89,90^. We also highlight that verbatim transcription has its own clinical merit, as disfluencies on their own are informative of language disorders^12,13,15,66^. Automatic transcription of non-fluent speech as well as replacing manual coding with machines would significantly benefit the field. We leave that for future endeavors.

## Conclusion

LLMs are increasingly transforming the field of NLP. However, it remains relatively understudied what a clinically accessible and interpretable LLMs-based NLP pipeline adds, and how it could advance automatic language analysis in language disorders such as aphasia. This study attempts to bridge the gap. We developed and evaluated such a pipeline, showcasing LLMs-surprisals as a diagnostic and predictive tool, and that pre-trained LLMs have great potential in generating meaningful language features without costly pre-processing, manual annotation, or sophisticated fine-tuning. Such features were statistically correlated with aphasia severity as well as the existing clinical language indices of aphasia. Adding the LLM features improves the models’ efficacy in predicting presence, subtypes, and severity of aphasia. We hope our investigation will lead to more nuanced questions on pinpointing NLP’s role in clinical research.

## Data availability

All the data in this study are drawn from the AphasiaBank (https://talkbank.org/DB/#), MacWhinney, B., Fromm, D., Forbes, M., & Holland, A. (2011). AphasiaBank: Methods for studying discourse. *Aphasiology*, 25, 1286–1307. The script for the analysis in this paper is available online: 10.17605/OSF.IO/KSV7P.

### Supplementary Information

Supplementary Information.
