# Supplementary material for: Clinical efficacy of pre-trained large language models through the lens of aphasia
Source: Sci Rep. 2024 Jul 6;14:15573. doi: 10.1038/s41598-024-66576-y (PMC11227580; doi:10.1038/s41598-024-66576-y)
Supplement: Supplementary file 1 — Supplementary Information. [file 41598_2024_66576_MOESM1_ESM.pdf]

Clinical efficacy of pre-trained Large Language Models through the lens of Aphasia

Supplementary information

	**Control (N=16791)**	**Aphasia (N=16791)**	**Total (N=33582)**	***p* value**
**age**				< 0.001
Mean	50.92	60.17	55.58	
Range	18.00 - 89.00	30.00 - 91.00	18.00 - 91.00	
SD	21.38	10.95	17.57	
**sex**				< 0.001
female	11284 (67.2%)	7235 (43.1%)	18519 (55.1%)	
male	5507 (32.8%)	9556 (56.9%)	15063 (44.9%)	
**WAB-R-AQ**				
Mean	NA	68.82	68.82	
Range	NA	10.80 - 92.80	10.80 - 92.80	
SD	NA	17.64	17.64	

Supplementary Table S1 Demographics information of dataset for aphasia versus healthy control classification task (N = number of utterances).

	**Total (N=6600)**
**age**	
Mean	61.01
Range	30.00 - 91.00
SD	8.98
**sex**	
female	885 (13.4%)
male	5715 (86.6%)

**WAB-R-AQ**	
Mean	63.47
Range	10.80 - 92.80
SD	19.38
**Subtype**	
Anomic	2200 (33.3%)
Broca	2200 (33.3%)
Wernicke	2200 (33.3%)

Supplementary Table S2 Demographics information of dataset for aphasia subtypes classification task (N = number of utterances).

ML Model	Grid search space
Decision tree	The function to measure the quality of a split (criterion): [gini, log_loss, entropy]<br>The maximum depth of the tree (max_depth): [2,4,6,8,10,12]<br>The number of features to consider when looking for the best split (max_features): [2,3,4,5] for LLMs model, [3,6,9,12] for the existing features model, and [3,6,9,12,15,17] for the model combining both LLMs and existing features
Random Forest	The function to measure the quality of a split (criterion): [gini, log_loss, entropy]<br>The number of trees (n_estimators): [100,200,300,400]<br>The number of features to consider when looking for the best split (max_features): same as Decision tree
Gradient boost	The function to measure the quality of a split (criterion): [friedman_mse, squared_error]<br>The number of boosting stages to perform (n_estimators): [100,200,300,400]<br>The number of features to consider when looking for the best split (max_features): same as Decision tree
SVM	Regularization parameter (C): [0.1, 1, 10, 100, 1000]<br>Kernel coefficient (gamma): [1, 0.1, 0.01, 0.001, 0.0001]<br>Kernel type to be used in the algorithm (kernel): [rbf, sigmoid]

Supplementary Table S3 Machine learning (ML) classifiers (aphasia versus healthy control) hyper-parameter tuning: grid search space

ML Model	Hyper parameters selected after tuning

Decision tree	criterion: gini, max_depth: 4, max_features: 3
Random Forest	criterion: gini, max_features: 3, n_estimators: 200
Gradient boost	criterion: friedman_mse, max_features: 3, n_estimators: 100
SVM	With all five LLMs features: C: 10, gamma: 0.1, kernel: rbf<br>With one LLM feature (Mistral) at a time: C: 1000, gamma: 0.01, kernel: rbf<br>With one LLM feature (GPTNeo2B7) at a time: C: 100, gamma: 1, kernel: rbf<br>With one LLM feature (GPTNeo1B3) at a time: C: 1, gamma: 1, kernel: rbf<br>With one LLM feature (DistilGPT2) at a time: C: 10, gamma: 0.1, kernel: rbf<br>With one LLM feature (GPT2) at a time: C: 100, gamma: 0.1, kernel: rbf

Supplementary Table S4 Machine learning classifiers (aphasia versus healthy control) hyper-parameter selected after tuning.

ML Model with the existing features	Hyper parameters selected after tuning
Decision tree	criterion: entropy, max_depth: 8, max_features: 3
Random Forest	criterion: gini, max_features: 9, n_estimators: 200
Gradient boost	criterion: friedman_mse, max_features: 9, n_estimators: 200
SVM	C: 1, gamma: 0.1, kernel: rbf

Supplementary Table S5 Machine learning classifiers (aphasia subtypes) hyper-parameter selected after tuning.

ML Model with the LLMs features	Hyper parameters selected after tuning
Decision tree	criterion: gini, max_depth: 4, max_features: 4
Random Forest	criterion: gini, max_features: 2, n_estimators: 300
Gradient boost	criterion: friedman_mse, max_features: 5, n_estimators: 200
SVM	C: 1000, gamma: 0.1, kernel: rbf

Supplementary Table S6 Machine learning classifiers (aphasia subtypes) hyper-parameter selected after tuning.

ML Model with the existing and LLMs features	Hyper parameters selected after tuning
Decision tree	criterion': 'entropy', 'max_depth': 2, 'max_features': 17
Random Forest	criterion': 'entropy', 'max_features': 6, 'n_estimators': 100
Gradient boost	criterion': 'friedman_mse', 'max_features': 3, 'n_estimators': 200
SVM	C': 1, 'gamma': 0.1, 'kernel': 'rbf'

Supplementary Table S7 Machine learning classifiers (aphasia subtypes) hyper-parameter selected after tuning.

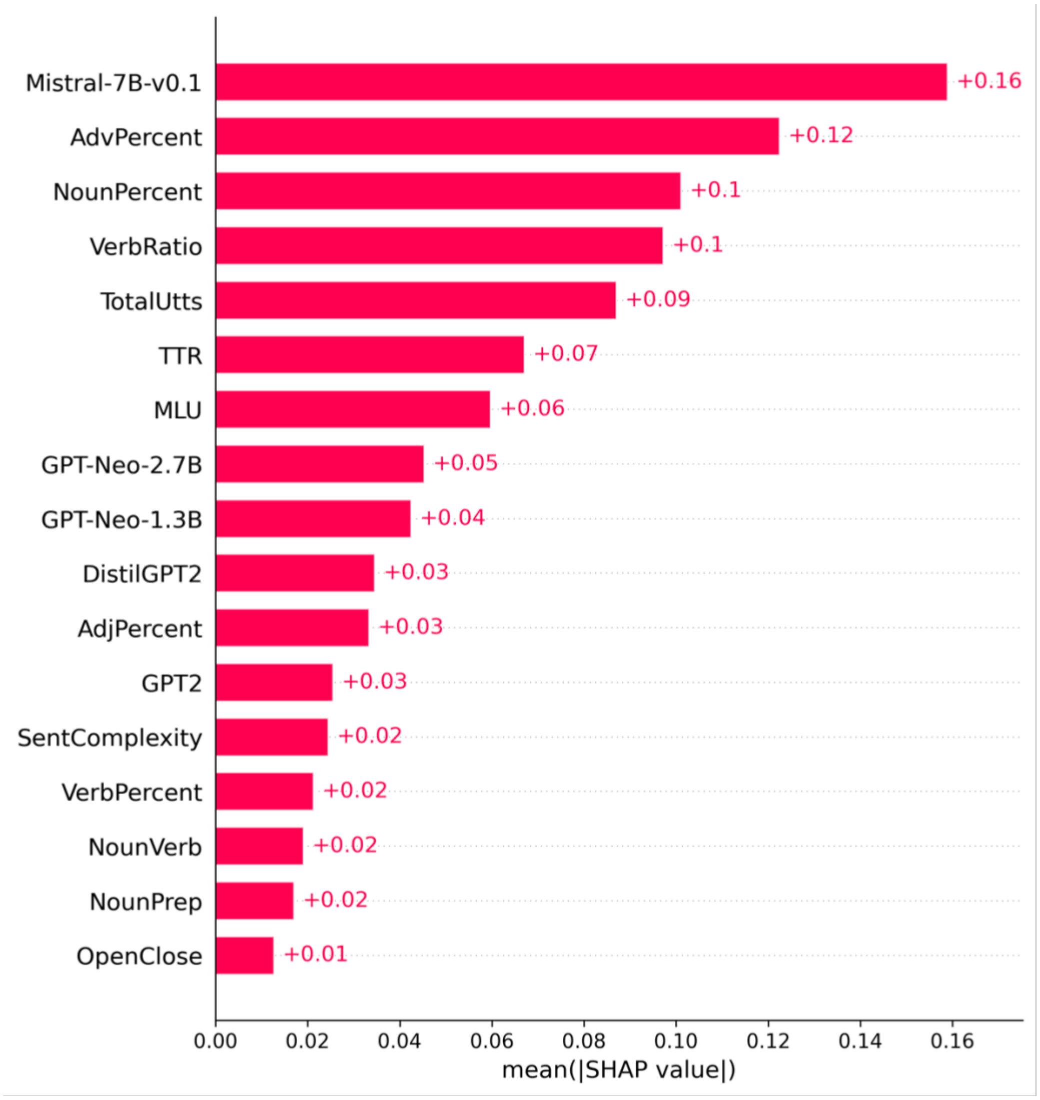

Supplementary Figure S1 Complete LLMs and existing features importance on predicting the subtypes of aphasia, rank based on the absolute SHAP value.
